# Linking Memory Impairment to Structural Connectivity in Extrahippocampal Temporal Lobe Epilepsy Surgery

**DOI:** 10.3390/neurolint17040052

**Published:** 2025-03-31

**Authors:** Niels Alexander Foit, Karin Gau, Alexander Rau, Horst Urbach, Jürgen Beck, Andreas Schulze-Bonhage

**Affiliations:** 1 Department of Neurosurgery, Faculty of Medicine, Medical Center, University of Freiburg, 79106 Freiburg, Germany; 2 Neuroimaging of Epilepsy Laboratory, McConnell Brain Imaging Center, Montreal Neurological Institute, McGill University, Montreal, QC H3A 2B4, Canada; 3 Department of Neuroradiology, Faculty of Medicine, Medical Center, University of Freiburg, 79106 Freiburg, Germany

**Keywords:** temporal lobe epilepsy, hippocampus, surgery, connectome, memory

## Abstract

Objective: Temporal lobe epilepsy (TLE) constitutes the most common drug-refractory epilepsy syndrome. Tailored approaches are required, as TLE originates from extrahippocampal lesions in about one-quarter of surgical candidates. Despite high success rates in seizure control, concern persists regarding postoperative memory decline after lesionectomy. We investigated the associations between structural connectivity and postoperative memory performance in extrahippocampal TLE surgery. Methods: In total, 55 patients (25 females, 30 males; mean age 29.8 ± 14.5 years; epilepsy duration 7.9 ± 10.5 years, 31 left, 24 right TLE) with extrahippocampal TLE undergoing hippocampal-sparing surgery were evaluated with standardized pre- and postoperative neuropsychological testing. Lesion volumes intersected with Human Connectome Project-derived tractography data were employed to assess the structural connectivity integrity via voxel-based and connectome-informed lesion–symptom mapping to identify cortical and white matter structures associated with cognitive outcomes. Results: Post-surgery, the widespread structural disconnection of several major white matter pathways was found, correlating with verbal memory and delayed recall. Additionally, the structural disconnection of the ipsilateral temporal lobe white matter was further associated with hippocampal atrophy. Conclusions: Our study highlights the role of structural connectivity alterations in postoperative memory decline in extrahippocampal TLE surgery. These findings expand the traditional understanding of hippocampal integrity in memory function towards the importance of broader structural networks. Individualized, connectome-informed surgical approaches might protect neurocognitive function.

## 1. Introduction

Epilepsy affects nearly 1% of the world’s population, with hippocampal sclerosis [1] representing the most frequent histopathological finding in mesial temporal lobe epilepsy (TLE). However, in approximately one-quarter of all TLE patients, seizures originate from extramesial lesions [2,3,4]. Magnetic resonance imaging (MRI) has been pivotal for lesion identification [5], while other modalities such as magnetoencephalography or invasive recordings complement clinical decision-making in MRI-negative patients, streamlining access to epilepsy surgery [6]. In this TLE subtype, a normal-appearing hippocampus on MRI has been considered structurally and functionally intact [7,8,9,10], promoting the development of surgical techniques to prevent memory decline [11,12,13]. While temporal lobe surgery leads to seizure freedom for many patients, it poses a significant risk of postoperative memory impairment [11,14]. Notably, growing evidence now suggests that even lesionectomy only partially shields patients from neurocognitive decline [7,14]. In our previous work, we demonstrated that extrahippocampal resections led to ipsilateral hippocampal atrophy and postoperative memory impairment, despite hippocampal sparing [15]. Larger resection volumes led to increased atrophy and the pronounced impairment of memory networks [16,17]. It is therefore conceivable that even limited resections in extramesial TLE negatively impact memory networks. Several studies in TLE have focused on the relationships between structural network integrity and memory performance [9,18,19,20]. Diffusion tensor imaging (DTI) studies demonstrated post-surgical widespread alterations in white matter (WM) microstructure adjacent [21,22] and distant from the resection [18,23], which has recently been confirmed in a post-mortem fiber dissection study [24]. Moreover, advances in high-resolution in vivo imaging now allow for an increasingly detailed characterization of the hippocampal connectome [25,26], revealing hippocampal WM projections disconnected following epilepsy surgery [20,21,27]. It seems plausible that the structural disconnection of WM pathways therefore contributes to neurocognitive decline [20,22], with hippocampal atrophy representing a subsequent structural correlate of disconnection [28,29,30,31]. Despite these advances, our current understanding of the dynamics of memory networks after hippocampus-sparing surgery remains limited. We therefore investigated the structural connectivity and memory performance in hippocampus-sparing TLE surgery. We hypothesized that hippocampal atrophy and memory impairment represent the clinical phenotype of direct and indirect structural disconnection (SDC) within a distributed network. Thus, the impact of a lesion on the structural connectome likely represents a crucial determinant of behavioral effects [32] rather than focal effects on critical gray matter (GM) regions alone [8]. To identify structures driving this clinical phenotype, a two-step approach was employed, i.e., (a) classical voxel-based lesion–symptom mapping (VLSM) using cortical parcellations and (b) connectome-informed lesion–symptom mapping. By evaluating structural connectivity alterations without any a priori assumptions, we evaluated the potential relationships with postoperative memory performance in a purely data-driven analysis [33].

## 2. Materials and Methods

### 2.1. Participants

**Figure 1:**
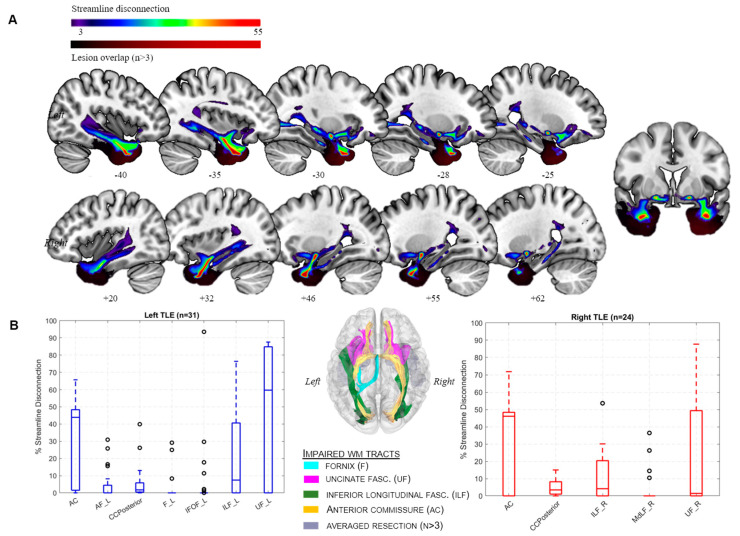
White matter tracts and structural disconnection. (**A**) Individual tract density volumes (*n* = 54) relative to HCP842 after hippocampus-sparing temporal lobe resection (31 left), overlaid on the MNI152 template. Color bar indicates overlap of disconnected white matter (WM) streamlines (spectrum) and surgical lesion (black–red), respectively. Figure orientation follows neurological convention (left side of image is left), and MNI z coordinates of sagittal sections are reported. (**B**) Tract disconnection severity. Disconnections in major WM tracts include the anterior commissure (AC), bilateral uncinate fascicle (UF), bilateral inferior longitudinal fasciculus (ILF), posterior portion of the corpus callosum (CCposterior), and left arcuate fasciculus (AF). Mean, standard deviations, and % disconnection of canonical WM tracts from the HCP842 dataset are given. For orientation purposes, affected canonical tracts are visualized on a transparent MNI-152 T1w surface, including an averaged surgical resection volume (*n* > 3, center).

We studied 55 patients with drug-refractory, extrahippocampal TLE (25 females, 30 males; mean age 29.8 ± 14.5 years; epilepsy duration 7.9 ± 10.5 years, 31 left, 24 right TLE) from a previously published cohort [15]. Briefly, definitive diagnosis and lateralization of epilepsy were determined by comprehensive presurgical investigations, including detailed history, neurological examination, MRI assessment, and surface or invasive video-EEG monitoring, confirming unilateral seizure onset within the temporal lobe. Importantly, all hippocampi of the study group were classified as normal-appearing on clinical MRI by experienced board-certified neuroradiologists [15]. All participants underwent standardized neuropsychological evaluation, i.e., including tests of verbal memory (verbal learning and memory test, VLMT) [34] and figural learning (DCS-R) [35]. Raw test scores were transformed into z-scores according to normative data [36]. Postoperative seizure outcome was determined according to the modified Engel classification [37]. All patients underwent tailored lesionectomy with sparing of the hippocampal formation [15], resulting in freedom from seizures, i.e., Engel class I outcome in 38 patients (69.1%). Resections of the epileptogenic focus were mainly guided by the presence of structural lesions on preoperative MRI. In case of discordant electroclinical findings or unrevealing imaging, surgical strategies were complemented by invasive recordings or intraoperative electrocorticography. For an overview of anatomical resection locations and extent, see Figure 1. Further detailed clinical and demographical data of the cohort are provided in Table 1 and in our previous work [15].

### 2.2. MRI Data Acquisition and Preprocessing

Details of imaging data collection, processing and standardization have previously been described [15]. In brief, high-resolution T1-weighted isotropic MRI data were obtained preoperatively and postoperatively (5.0 ± 4.0 months) and underwent standardized preprocessing and registration in the Montreal Neurological Institute (MNI) 152 template in SPM8 (https://www.fil.ion.ucl.ac.uk/spm/software/spm12/, accessed on 2 July 2021), run in MATLAB (R2019b; MathWorks, Natick, MA, USA). Resection volumes were derived from postoperative T1-weighted imaging by means of user-guided active contour segmentation in ITK Snap (version 3.4) [38] and normalized to MNI-152 template space using default procedures in SPM8. Hippocampal volume was estimated using atlas-derived regions of interest (ROIs), as previously detailed [15].

### 2.3. Lesion–Symptom Mapping

We evaluated associations between memory performance and markers of structural connectome impairment, i.e., region and WM disconnection as well as HC atrophy without any a priori assumptions, i.e., allowing for purely data-driven analyses [33].

Atlas-based VLSM. Whole-brain VLSM [39] was performed using Niistat [https://www.nitrc.org/projects/niistat/] (accessed on 2 July 2021). Cortical parcellations of 192 homotopic areas were derived from the AICHA atlas of intrinsic connectivity [40]. This approach addresses multiple testing issues involved in large voxel-wise statistics [41] by effectively reducing dimensionality through incorporating a priori information on regional boundaries [42,43,44], thus allowing the lesion data to be represented by a limited number of known anatomical structures [42].

### 2.4. Structural Connectome Parameterization

Lesion volumes aligned with MNI-152 space were analyzed with the Lesion Quantification Toolkit by Griffis and co-workers [42], estimating SDC parameters of WM and between ROIs, using an endpoint-based criterion for identifying structural disconnection. This facilitates combining a functional imaging-derived brain atlas [45] with high-resolution tractography data [46] for a comprehensive, anatomically informed description of lesions’ effects on the structural connectome [19,43,47].

White matter disconnection. Impaired WM structural connectivity was evaluated on a macroscale level of 70 canonical WM tracts [19,26] by means of tract-based disconnection, a common approach in TLE research [18,19,21]. Voxel-wise tract density image (TDI) estimates were obtained for all canonical WM tracts included in the Human Connectome Project (HCP)-842 tractography atlas [46]. As a first step, the surgical lesion volumes were embedded into the aggregate HCP-842 tractography atlas as ROIs, followed by iterative filtering, i.e., retaining only streamline trajectories intersecting the lesion volume. This results in an estimate of % disconnection of the respective canonical tract [42,48]. Notably, tract-based severity estimates provide a biologically more meaningful representation of impaired neural signal transmission compared to lesion load [49] or probability-based approaches [42,50].

Parcel-wise disconnection. In addition to tract-based analyses, we further evaluated cortical connectivity based on whole-brain direct region-to-region structural connections, i.e., parcel-wise disconnection induced by the resection. To evaluate parcel-wise disconnection severity, a structural connectome was modeled by combining HCP842-derived streamline trajectories [46] with a cortical parcellation, i.e., 135 distinct brain from the extended Schaefer–Yeo atlas [42,45,48]. Following connectivity matrix construction and lesion volume embedding, streamline trajectories were iteratively filtered, retaining only those intersecting both the lesion (i.e., disconnected streamlines) and terminating bilaterally within a pair of cortical areas [32]. This results in a percent disconnection severity matrix relative to the atlas structural connectome, allowing for a detailed representation of connectivity alterations caused by the specified lesion, specifically, resected brain volume [42].

### 2.5. Statistical Analysis

Atlas-based lesion–symptom mapping. Univariate general linear models (GLMs), i.e., pooled-variance t-tests and linear regression were used to compare memory performance in patients with lesional versus non-lesional voxels, present in at least three patients. Lesion volume was regressed out during permutation testing [51]. Resulting *beta*-maps underwent permutation testing with 5000 permutations, with results surviving family-wise error (FWE)-corrected thresholds of *p* < 0.05 considered statistically significant [52].

Structural connectome. Relations between WM disconnection and behavioral test data were evaluated with publicly available Matlab scripts, harnessing advantages of general linear models (GLMs) combined with rigorous permutation testing. In this mass-univariate approach, possible linear relations of the respective behavioral variables, i.e., VLMT z-scores, with individual structural lesion loads for each canonical WM tract or connections between brain regions of the structural connectome are investigated. In-depth information on methodological considerations and respective processing steps have previously been detailed and are available from the original work of Sperber and Anziano [41,47]. Briefly, a mass-univariate GLM is computed and conservative “maximal statistic permutation testing” significance thresholds for the estimated parameters are applied by means of permutation testing, followed by FWE correction between pairs of cortical parcels, WM tracts, and VLMT z-scores, respectively. Since disconnections are expected to elicit deficits only, all results are reported as one-tailed tests at FWE-corrected thresholds of *p* < 0.05, with 5000 permutations applied [53]. Only disconnections present in *n* > 3 patients were considered relevant.

## 3. Results

Atlas-based lesion–symptom mapping. The VLSM revealed significant associations between decreased verbal learning performance and lesion data in the left inferior frontal cortex (cluster size 45 voxels, *t* = 2.39) and left parahippocampal gyrus (PHG, cluster size = 73 voxels, *t* = 2.70). There were no significant voxel clusters associated with reduced visual memory performance or hippocampal volume.

White matter tract disconnection. Extensive streamline disconnection was found in 5 of 70 canonical tracts in left HC-sparing resections (Figure 1A), i.e., the anterior commissure (AC), inferior longitudinal (ILF), uncinate (UF), inferior fronto-occipital (IFOF), arcuate fascicle (AF), posterior portion of the corpus callosum (CCPosterior), and left fornix (F). The right-hemispheric resections resulted in similar streamline disconnection patterns, including the AC, UF, ILF, and CCPosterior (Figure 1B). With regard to verbal memory, the structural disconnection of two important WM tracts, i.e., the left fornix (t = 2.9/slope = 1.9) and IFOF (t = 5.8/slope = 4.3), was associated with impaired memory performance (Table 2). Moreover, the structural disconnection of two large WM tracts, i.e., IFOF (t = 2.7, r^2^ = 0.14, slope = 1.04/) and ILF (t = 2.9/r^2^ = 0.14/slope = 1.66), were significantly associated with ipsilateral HC atrophy while the UF missed significance levels (t = 2.4/slope =1.9/r^2^ = 0.11). The correlation coefficients were indicative of moderate effect sizes. There was no significant association between visual memory, i.e., DCS scores, and large WM tracts.

**Figure 2:**
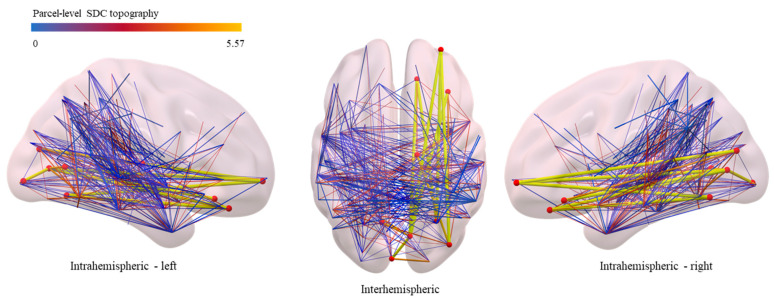
Region-to-region structural disconnections. Group-level topography of significant region-to-region structural disconnections correlating with delayed verbal recall. Significant nodes, i.e., cortical areas (red volumes) affected by WM disconnection (edges, blue–yellow) are overlaid onto a smoothed MNI-152 volumetric template. Extensive disconnections within the ipsilateral temporal lobe and orbitofrontal, limbic, and visual areas, extending to the contralateral hemisphere, are found.

Region-to-region disconnection. Structural connectivity was disrupted in eight distinct ROI-to-ROI connections (1115 pairs of cortical parcels analyzed, *n* > 3 patients affected). The inter-regional disconnections are visualized in Figure 2. A decreased delayed verbal recall performance (VLMT subtest 5–7) correlated with a distributed multi-node network, connecting parcels of the right hemisphere, i.e., orbitofrontal cortex, prefrontal cortex, and visual and subcortical areas as well as the contralateral hemisphere (Figure 2), indicative of widespread alterations distant to the lesional area.

## 4. Discussion

We evaluated the impact of extrahippocampal lesionectomy in TLE patients on the integrity of the structural connectome. Expectedly, lesionectomy within the temporal lobe resulted in the widespread structural disconnection of WM tracts, extending beyond the resection site; disconnection additionally correlated with ipsilateral hippocampal volume loss. Associations between the neurocognitive performance, and particularly the verbal memory, and disconnected major WM tracts were identified, suggesting that, despite resulting in excellent seizure control, tailored resections in the temporal lobe could promote neurocognitive decline through structural WM impairment, which has previously not been captured by atlas-based or volumetric studies [26,54,55]. Consequently, the measures of disconnection severity and region-to-region connectivity allowed for a better characterization of distributed WM network disruption [32,48], which could be missed by utilizing classic approaches. Our findings further corroborate evidence from patients with hippocampal sclerosis undergoing anterior temporal lobe resection (ATL), exhibiting equally widespread WM impairment and reduced language and memory performance [23,56,57]. It seems therefore plausible that even limited temporal lobe resections disrupt large-scale networks, with atrophy occurring at a distance from the resection site due to the loss of connectivity between affected brain regions. Comparing selective surgery vs. ATL, Arnold and co-workers have recently confirmed cortical thinning in the ipsilateral insula, temporal lobe, and even contralateral hippocampus, with larger resections leading to more pronounced atrophy. These findings could represent a structural analog for functional connectivity observations [58]. Our study further adds to evidence from low-grade glioma resections and stroke, highlighting the dependence of recovery from cortical insults on preserved WM tracts [22]. Since extrahippocampal TLE frequently stems from cortical lesions [7,11], minimally invasive procedures such as interstitial thermotherapy could harbor potential for the preservation of neurocognitive functioning compared to resective surgery [20,59,60], achieving favorable seizure control in up to two-thirds of all candidates [61]. Despite these encouraging results, standard resective procedures still achieve slightly higher rates of freedom from seizures [62]. However, growing evidence now suggests that network- and connectome-derived biomarkers harbor further potential to further improve seizure control rates in minimally invasive procedures. Two recent studies have indeed demonstrated that individual variations in the structural connectome or its topography were highly predictive of surgical success rates, surpassing traditional clinical prediction models [63,64]. In this regard, the integration of novel biomarkers into the preoperative workflow could ultimately improve the surgical precision of tailored approaches [65,66].

This tentative concept nevertheless requires validation in larger cohorts. We further identified several WM tracts and parcel-wise disconnection patterns associated with verbal memory decline. This effect was not unexpected, since both PHG and fornix constitute core structures of the limbic memory network, with the latter representing its primary projection tract [26,67]. Altered fornix connectivity was nevertheless unexpected, since the fornix originates from the most mesial temporal structures, while resections were mainly cortical. Nevertheless, there is growing evidence that the transection of WM pathways during epilepsy surgery could affect structures at a distance or even contralaterally [31,68]. In particular, in the context of postoperative hippocampal atrophy, the obtained disconnection measures of the fornix could reflect WM degeneration as a secondary structural correlate of atrophy instead of direct surgical damage [69,70].

Importantly, two major temporal lobe WM tracts, i.e., ILF and IFOF, were found to be associated with postoperative hippocampal atrophy, corroborating previous observations in TLE surgery [15,31,58,71]. Importantly, since the ILF has recently been identified as a distinct hippocampal projecting pathway by means of super-high-resolution diffusion-weighted imaging in vivo [26], it seems plausible that a structurally impaired ILF deprives the HC of sensory information input, hindering memory formation and retrieval [72] with subsequent hippocampal atrophy [71,73]. Further evidence from TLE highlights the importance of the temporal neocortex and WM for the preservation of neurocognitive functioning [20,27]. However, this plausible hypothesis requires further validation in larger cohorts as well. Although the IFOF does not directly share projections with the hippocampus [26], it is nevertheless part of a distributed network connecting regions involved in visual, cognitive, and memory functions [22,74]. These findings are in line with growing evidence that memory and language function indeed depend on a more widely distributed network [27,75]. It seems likely that such broader WM disruptions could equally impair memory processing on larger scales and contribute to hippocampal atrophy, which, in turn, emphasizes the importance of distributed WM networks for preserved neuropsychological functioning [23,27,74]. In this regard, Kaestner and co-workers recently identified an impaired WM microstructure as an independent predictor of postoperative memory decline following ATL [20]. Finally, although the anterior commissure was found to exhibit significant reductions in streamline density (Figure 1), we did not find any associations with memory performance. Nevertheless, these results are in line with those of other studies indicating that temporal lobe resections can promote structural alterations in this major WM tract.

There are several limitations to our study, which mainly pertain to the retrospective data collection and sample size. Due to the retrospective nature, we only assessed verbal memory and WM alterations after approximately 6 months, which does not allow for long-term predictions. Nevertheless, evidence from longitudinal studies indicates that both verbal memory impairment and WM alterations evolve significantly beyond the initial postoperative period [58,69]. Importantly, patients could experience a progressive decline in verbal memory function for up to two years post-surgery, after which this decline tends to stabilize [21,23]. These findings underscore the dynamic nature of cognitive functions and WM integrity following TLE surgery, which should be explored in future longitudinal work. In this regard, our sample was too small to further elucidate potential differences in structural connectome characteristics between Engel class I individuals and patients with unfavorable seizure outcomes. Further, our study may be underpowered to detect discrete differences in structural alterations of smaller WM tracts or parcel-wise disconnections. Nevertheless, our findings survived robust FWE correction. A minor limitation pertains to the lack of individual diffusion-weighted MRI data, necessitating structural disconnection evaluation through lesion embedding with a high-resolution structural connectome atlas [46]. This approach is nevertheless commonly used in lesion–symptom studies [19,32,43,48,50]. While clearly not accounting for interindividual variations in undamaged WM, it effectively avoids the detrimental impact of variable diffusion MRI acquisition quality in group studies, particularly for postoperative patients [42]. Furthermore, we utilized an ultra-high-resolution tractography atlas derived from a very large homogenous sample, reducing the likelihood of false-positive fiber tract reconstructions [46].

## 5. Conclusions

This study confirms associations between WM disconnection following lesionectomy for extrahippocampal TLE, hippocampal atrophy, and neurocognitive decline, particularly in verbal memory domains. Our findings further emphasize the distributed nature of memory networks and confirm the utility of modern connectome-derived biomarkers compared to traditional volumetric approaches. By modeling resection effects on structural connectivity and relating them to postoperative neurocognitive markers, we can advance our understanding on the effects of local treatments affecting the connectome on larger scales. Importantly, although tailored resections often achieve effective seizure control, their potential impact on neurocognitive outcomes calls for minimally invasive alternatives, which may preserve connectivity. Further validation in larger cohorts is needed to create predictive models for cognitive outcomes.

## Figures and Tables

**Table 1:** Demographic and clinical information.

Participant	Sex	Side of Surgery	Age at Surgery	Seizure Onset	Duration	Tailoring	Histopathology
			[Years]	[Years]	[Years]	[Modality]	
1	male	right	20	16	4	MRI	Nonspecific alterations, gliosis
2	female	left	22	16	6	ECOG	Ganglioglioma II°
3	female	right	6	3	3	ECOG	Malformation of cortical development
4	female	right	28	17	11	sEEG / ECOG	Malformation of cortical development
5	male	left	25	18	7	sEEG	Cortical gliosis, nonspecific alterations
6	female	right	45	27	18	ECOG	Malformation of cortical development
7	male	left	18	17	1	MRI	DNT I°
8	female	right	42	35	7	MRI	Ganglioglioma I°
9	male	left	16	16	0	MRI	Ganglioglioma I°
10	male	left	35	25	10	MRI	Nonspecific alterations
11	female	left	6	5	1	MRI	Ganglioglioma I°
12	male	left	55	54	1	MRI	Cavernoma
13	female	left	13	13	0	MRI	Piloytic astrocytoma
14	male	left	31	13	18	sEEG / ECOG	Malformation of cortical development
15	male	left	26	24	2	MRI	Ganglioglioma I°
16	female	left	43	39	4	MRI	Cavernoma
17	male	right	8	7	1	MRI	Piloytic astrocytoma
18	female	right	26	21	5	ECOG	Nonspecific alterations
19	male	right	20	19	1	MRI	Ganglioglioma I°
20	male	left	17	14	3	MRI	Pilocytic astrocytoma I°
21	female	left	21	20	1	MRI	Cavernoma
22	female	left	42	31	11	MRI	Cavernoma
23	male	left	45	39	6	MRI	Xanthoastrocytoma II°
24	male	right	42	24	18	ECOG	Malformation of cortical development
25	female	left	56	27	29	MRI	Vascular lesion
26	male	right	15	6	9	ECOG	Malformation of cortical development
27	male	right	33	23	10	sEEG	Malformation of cortical development
28	male	left	24	14	10	MRI	Malformation of cortical development
29	female	left	60	48	12	sEEG	No definitive histopathology
30	female	right	19	18	1	MRI	Xanthoastrocytoma II°
31	female	left	16	12	4	ECOG	Malformation of cortical development
32	male	left	38	37	1	MRI	Ganglioglioma I°
33	male	right	60	58	2	MRI	Cavernoma
34	female	left	27	26	1	MRI	Ganglioglioma I°
35	female	left	38	16	22	MRI	Cavernoma
36	female	right	46	45	1	MRI	Cavernoma
37	male	right	9	8	1	MRI	Malformation of cortical development
38	female	left	24	20	4	MRI	Cavernoma
39	male	right	43	40	3	MRI	Malformation of cortical development
40	male	right	40	36	4	MRI	Ganglioglioma I
41	male	left	45	16	29	ECOG	Nonspecific alterations
42	female	right	11	10	1	MRI	DNT I°
43	male	right	37	32	5	MRI	Cavernoma
44	male	left	18	11	7	sEEG	Malformation of cortical development
45	male	right	58	9	49	MRI	Ganglioglioma I°
46	male	left	24	18	6	MRI	Nonspecific alterations
47	female	left	38	34	4	MRI	Encephalocele
48	male	right	18	18	0	MRI	Ganglioglioma
49	male	right	51	2	49	MRI	Malformation of cortical development
50	female	right	26	24	2	MRI	Malformation of cortical development
51	female	right	29	20	9	MRI	DNT I°
52	female	left	17	12	5	MRI	No histopathology available
53	male	left	23	20	3	MRI	Nonspecific alterations
54	female	left	26	14	12	MRI	Cavernoma
55	male	left	19	17	2	MRI	Malformation of cortical development

Demographic and clinical data of the study cohort. MRI—magnetic resonance imaging; DNT—dysembryoblastic neuroepithelial tumor; sEEG—stereoelectroencephalography; ECOG—electrocorticography.

**Table 2:** Tract disconnection results.

Tract	Disconnection	Neurocognitive Performance/Hippocampal Volume			
	Median % disconnection ± SD	Behavioral test	t (FWE)	r^2^	Slope
Fornix (L)	5.8 ± 11.4	VLMT	2.88	0.26	1.90
IFOF (L)	5.0 ± 17.6	VLMT	5.57	0.31	3.10
		HC volume	2.7	0.14	1.04
ILF	N/A	HC volume	2.9	0.14	1.66

Tract disconnection results. Mean percentage of streamline disconnections in major and canonical white matter pathways (left panel) and their respective associations with verbal memory and hippocampal volume reduction (right panel). IFOF—inferior frontooccipital fascicle; ILF—inferior longitudinal fascicle; HC—hippocampus; VMLT—verbal learning and memory test.

## Data Availability

Clinical records are not made publicly available since they contain sensitive information that could compromise privacy of the research participants, but are available from the authors upon reasonable request. All Matlab scripts for connectivity-informed analyses are available from the authors upon request.
